# Characterization of a *Streptococcus mutans* Intergenic Region Containing a Small Toxic Peptide and Its *cis*-Encoded Antisense Small RNA Antitoxin

**DOI:** 10.1371/journal.pone.0054291

**Published:** 2013-01-11

**Authors:** Stephanie Koyanagi, Céline M. Lévesque

**Affiliations:** Dental Research Institute, Faculty of Dentistry, University of Toronto, Toronto, Ontario, Canada; University of Kansas Medical Center, United States of America

## Abstract

Toxin-antitoxin (TA) modules consist of a pair of genes that encode two components: a protein toxin and an antitoxin, which may be in the form of either a labile protein or an antisense small RNA. Here we describe, to the best of our knowledge, the first functional chromosomal type I TA system in streptococci. Our model organism is the oral pathogen *Streptococcus mutans*. Our results showed that the genome of *S. mutans* UA159 reference strain harbors a previously unannotated Fst-like toxin (Fst-Sm) and its *cis*-encoded small RNA antitoxin (srSm) converging towards the end of the toxin gene in IGR176, a small intergenic region of 318 nt. Fst-Sm is a small hydrophobic peptide of 32 amino acid residues with homology to the Fst toxin family. Transcripts of ∼200 nt and ∼70 nt specific to *fst-Sm* mRNA and *srSm* RNA, respectively, were detected by Northern blot analysis throughout *S. mutans* growth. The toxin mRNA was considerably more stable than its cognate antitoxin. The half-life of *srSm* RNA was determined to be ∼30 min, while *fst-Sm* mRNA had a half-life of ∼90 min. Both *fst-Sm* and *srSm* RNAs were transcribed across direct tandem repeats providing a region of complementarity for inhibition of toxin translation. Overproduction of Fst-Sm had a toxic effect on *E. coli* and *S. mutans* cells which can be neutralized by coexpression of *srSm* RNA. Deletion of *fst-Sm*/*srSm* locus or overexpression of Fst-Sm/srSm had no effect on *S. mutans* cell growth in liquid medium and no differences in the total biofilm biomass were noted. In contrast, mild-overproduction of Fst-Sm/srSm type I TA system decreases the levels of persister cells tolerant to bacterial cell wall synthesis inhibitors.

## Introduction

Prokaryotic chromosomes contain small genetic elements encoding two components: a stable toxin and its less stable cognate antitoxin. These modules are called toxin-antitoxin (TA) systems [1]. Typically, the toxin inhibits an essential microbial cellular function. TA pairs form a stabilized complex in the cell preventing toxicity under normal growth conditions. However, when the balance between the toxin and antitoxin is perturbed, usually following cellular damage or stressful conditions, the toxin is released from the TA complex leading to cell growth arrest and/or cell death. The physiological significance and importance of TA modules in microbial physiology is reflected by the fact that they are ubiquitously found on the chromosome of bacteria [2]. Five different types of bacterial TA systems have been described thus far, depending of the nature and mode of action of the antitoxin component. While toxins are always proteins, antitoxins are either RNAs (type I, type III) or proteins (type II, type IV, type V). RNA antitoxins suppress toxin expression (type I) [3]–[5] or interfere with the toxin activity instead of preventing its expression (type III) [6], [7]. Protein antitoxins neutralize the toxicity of the cognate toxin by forming a stable complex (type II) [8]–[10], by functioning as antagonists for the toxin activity (type IV) [11], [12], or by inhibiting the toxin by cleaving specifically its mRNA (type V) [13]. Current hypotheses propose that TAs are stress-response elements that help bacteria cope with environmental stress either by promoting altruistic death of a subpopulation or by inducing formation of dormant persister cells [14]–[16]. The dormant state allows bacteria to survive even high doses of the antibiotic. It has been suggested that persister cells may play a significant role in the recalcitrance of chronic biofilm infections to antimicrobials [17]. In *E. coli*, multiple type II TA systems have been linked to the formation of persister cells [18], [19].

The oral cavity environment is inarguably a dynamic and complex ecosystem with a wide range of environmental challenges. Our model organism is the oral pathogen *Streptococcus mutans*. *S. mutans* depends on a biofilm lifestyle for its survival and persistence in its natural habitat [20]. In presence of fermentable dietary carbohydrates, this acid-producing bacterium can cause damage (cavities) to the tooth’s hard tissues [21], [22]. In fact, *S. mutans* is a major human pathogen that infects more than half of the world’s human population. We recently characterized a locus encoding a functional type II TA system, the *S. mutans* chromosomal MazEF module. We demonstrated that MazF protein was a toxic nuclease arresting cell growth through the mechanism of RNA cleavage, and that MazE antitoxin protein inhibited the ribonuclease activity of MazF by forming a protein complex [23]. Our most recent data showed that ectopic mild overexpression of chromosomal MazEF and RelBE type II TA systems induced formation of multidrug tolerant persister cells in *S. mutans* [24].

In this study, we are pursuing our investigation of the chromosomal TA systems in *S. mutans*. We were interested in discovering type I TA systems in the chromosome of *S. mutans*. In type I TA systems, antitoxins are small untranslated RNAs acting as antisense RNAs. RNA antitoxins can be encoded directly opposite the coding sequence of the toxin, opposite the 5′ UTR, or opposite the 3′ UTR of the toxin mRNA, or even divergent to the toxin gene but with long stretches of complementarity to the toxin mRNA [3]. The RNA antitoxins are acting as antisense RNAs that anneal with the corresponding toxin mRNA, thereby inhibiting its translation or promoting its degradation. All type I toxins are small, hydrophobic peptides of 19–38 amino acid residues. Overexpression of these hydrophobic toxins has been shown to lead to membrane depolarization or membrane disruption resulting in a loss of cell viability [3]. Type I TA modules were first discovered on plasmids where they were found to stabilize various plasmids in Gram-negative bacteria (post-segregational killing activity) [25]. The RNAI-RNAII encoded by the *par* locus of the *Enterococcus faecalis* plasmid pAD1 was the first type I TA system identified in Gram-positive bacteria. RNAI encodes a 33-aa peptide toxin (Fst), while RNAII codes for the ∼70-nt regulatory antisense RNA [26], [27]. The *par* addiction module kills plasmid-free cells by affecting membrane permeability and cell division [28], [29]. Recently, Weaver et al. (2009) [30] identified pAD1-like TA systems on the chromosomes and plasmids of *Enterococcus*, *Lactobacillus*, and *Staphylococcus* species suggesting that Fst-like toxin may be prevalent in Gram-positive bacteria.

Our results showed that the genome of *S. mutans* UA159 reference strain harbors an unannotated Fst-like toxin based on the presence of a conserved open reading frame. Examination of the genetic context revealed conserved features similar to the Fst type I TA system. The aim of this study was to investigate the functionality of this putative *S. mutans* type I TA system; we proposed the name Fst-Sm/srSm for Fst *S* *. mutans* and small RNA *S. mutans*. The possible link between Fst-Sm/srSm TA system and the development of dormant persister cells was also investigated. The Fst-Sm/srSm module is to our knowledge the first type I TA system characterized in *Streptococcus*.

## Materials and Methods

### Bacterial Growth Conditions

*S. mutans* strains were grown in Todd-Hewitt broth supplemented with 0.3% yeast extract (THYE) and incubated statically at 37°C in a 5% CO_2_ atmosphere. *E. coli* strains were cultivated aerobically in Luria-Bertani (LB) medium at 37°C. When needed, antibiotics were used as follows: chloramphenicol (20 µg/ml) or kanamycin (50 µg/ml) for *E. coli*, and erythromycin (10 µg/ml), or chloramphenicol (10 µg/ml) for *S. mutans*. Cell growth was monitored through optical density at 600 nm (OD_600_). Cell viability was assessed by counting CFU on replica agar plates.

### Plasmid and Strain Construction

A summary of bacterial strains and plasmids is provided in Table 1. Primers used for the generation of PCR products indicated below are listed in Table 2. A nonpolar insertion-deletion IGR176 mutant (ΔIGR176) was constructed in *S. mutans* UA159 wild-type (WT) strain by PCR ligation mutagenesis using the primer pairs CMT-576/CMT-577 and CMT-578/CMT-579. All plasmids were constructed in *E. coli* strain DH10B or TOP10. Plasmids were introduced into *E. coli* by transformation using electroporation or chemical transformation. Plasmids were transferred to *S. mutans* by natural transformation as described previously [31].

**Table 1:** Bacterial strains and plasmids used in this study.

Strain or plasmid	Relevant characteristic(s)^a^	Source or reference
Strains		
*S. mutans*		
UA159	Wild-type *S. mutans* reference strain	Lab stock
ΔIGR176 mutant	In-frame IGR176 deletion mutant derived from *S. mutans* UA159; Em^r^	This study
UA159(pIB166)	UA159 harboring pIB166; Cm^r^	[23]
ΔIGR176(pIB166)	ΔIGR176 harboring pIB166; Em^r^, Cm^r^	This study
ΔIGR176(pSK10)	ΔIGR176 harboring pSK10; Em^r^, Cm^r^	This study
*E. coli*		
DH10B	Host for cloning and plasmid production	Lab stock
TOP10	Host for cloning and plasmid production	Invitrogen
LMG194	Host strain for pBAD expression	Invitrogen
LMG194(pSK1)	LMG194 harboring pSK1; Km^r^	This study
LMG194(pSK2)	LMG194 harboring pSK2; Km^r^	This study
LMG194(pSK8)	LMG194 harboring pSK8; Km^r^	This study
DH10B(pSK3)	DH10B harboring pSK3; Cm^r^	This study
DH10B(pSK7)	DH10B harboring pSK7; Km^r^	This study
DH10B(pHSG299)(pSK3)	DH10B harboring pHSG299, pSK3; Km^r^ Cm^r^	This study
DH10B(pHSG299)(pSK4)	DH10B harboring pHSG299, pSK4; Km^r^ Cm^r^	This study
DH10B(pHSG299)(pSK5)	DH10B harboring pHSG299, pSK5; Km^r^ Cm^r^	This study
DH10B(pSK3)(pSK6)	DH10B harboring pSK3, pSK6; Cm^r^ Km^r^	This study
DH10B(pSK4)(pSK6)	DH10B harboring pSK4, pSK6; Cm^r^ Km^r^	This study
DH10B(pSK5)(pSK6)	DH10B harboring pSK5, pSK6; Cm^r^ Km^r^	This study
Plasmids		
pBAD202/D-TOPO	Expression vector linearized and topoisomerase-activated; Km^r^	Invitrogen
pHGS299	High-copy-number cloning vector; Km^r^	Takara Bio USA
pPROBE-NT’	Promoterless GFP vector; Km^r^	[32]
pIB166	*Streptococcus*-*E. coli* shuttle plasmid; Cm^r^	[33]
pSK1	*Fst-Sm* cloned under the control of *araBAD* promoter into pBAD202/D-TOPO; Km^r^	This study
pSK2	*Fst-Sm/srSm* cloned under the control of *araBAD* promoter into pBAD202/D-TOPO; Km^r^	This study
pSK3	GFP cassette cloned into pIB166; Cm^r^	This study
pSK4	P*_fst_*+DRI-II transcriptionally fused to *gfp* into pSK3; Cm^r^	This study
pSK5	P*_fst_* transcriptionally fused to *gfp* into pSK3; Cm^r^	This study
pSK6	*srSm* cloned into pHSG299; Km^r^	This study
pSK7	*Fst-Sm* G16A mutation into pSK1; Km^r^	This study
pSK8	*Fst-Sm* A11G/G16A mutation into pSK1; Km^r^	This study
pSK9	*fst-Sm* cloned under the control of its own promoter into pIB166; Cm^r^	This study
pSK10	*fst-Sm* and *srSm* cloned under the control of their own promoter into pIB166; Cm^r^	This study

a Em^r^, erythromycin resistance; Cm^r^, chloramphenicol resistance; Km^r^, kanamycin resistance.

**Table 2:** Primers used in this study.

Primer	Gene	Sequence (5′→3′)a,b	Purpose
CMT-576	*fst-Sm/srSm*	GCACAATGGGAATCTGGAAG	Gene deletion
CMT-577	*fst-Sm/srSm*	GGCGCGCC AGGCATGATTTCTTTATTCGCA	Gene deletion
CMT-578	*fst-Sm/srSm*	GGCCGGCC TATCATATTCTCCACAAACGATA	Gene deletion
CMT-579	*fst-Sm/srSm*	GACTTATGGTCATTTGGTTGC	Gene deletion
CMT-19	*erm*	GGCGCGCC CCGGGCCCAAAATTTGTTTGAT	Gene deletion
CMT-20	*erm*	GGCCGGCC AGTCGGCAGCGACTCATAGAAT	Gene deletion
CMT-499	*fst-Sm*	CACCATGTGGCATAATTTCTTTATATA	pBAD cloning
CMT-500	*fst-Sm*	ATCGTCGTCTTTCTTATCCA	pBAD cloning
CMT-581	*fst-Sm/srSm*	CACCAGTTGCCAAATGTGGCATAAT	pBAD cloning
CMT-582	*fst-Sm/srSm*	TTGACAAAACCAAAATAAAAAGA	pBAD cloning
CMT-621	*fst-Sm*	CGCACCAATTTTTGTTG**C**GATAGTGCTTGCGCTAT	Mutagenesis
CMT-622	*fst-Sm*	ATAGCGCAAGCACTATC**G**CAACAAAAATTGGTGCG	Mutagenesis
CMT-627	*fst-Sm*	CATAATTTCTTTATATATATCGTCG**G**ACCAATTTTTGTTGCGATAGTG	Mutagenesis
CMT-628	*fst-Sm*	CACTATCGCAACAAAAATTGGT**C**CGACGATATATATAAAGAAATTATG	Mutagenesis
CMT-449	*gfp*	CTCGAG GTCACGACGTTGTAAAACGAC	GFP reporter
CMT-450	*gfp*	CCGCGG CAGCTATGACCATGATTACGC	GFP reporter
CMT-596	*fst-Sm*	GAATTC GACTGAAGATGAATTTGAAAAC	GFP reporter; *fst-Sm*, *fst-Sm/srSm* expression
CMT-597	*fst-Sm*	GGTACC ATATATAAAGAAATTATGCCACA	GFP reporter
CMT-598	*fst-Sm*	GGTACC CACCCCCTCTCTAAGGCG	GFP reporter
CMT-599	*srSm*	GGATCC TTGACAAAACCAAAATAAAAAGA	GFP reporter; *srSm* expression
CMT-600	*srSm*	GAATTC TAGCTCGGACGCAGTATTC	GFP reporter; *srSm* expression
CMT-595	*fst-Sm*	AAGCTT ACTTAATCGTCGTCTTTCTTAT	*Fst-Sm* expression
CMT-594	*fst-Sm/srSm*	AAGCTT TTGACAAAACCAAAATAAAAAGA	*fst-Sm/srSm* expression
CMT-497	*fst-Sm*	ATGTGGCATAATTTCTTTATATA	RT-PCR, Northern
CMT-498	*fst-Sm*	TTAATCGTCGTCTTTCTTATCC	RT-PCR, Northern
CMT-583	*fst-Sm*	TTAATCGTCGTCTTTCTTATC	5′RACE-PCR
CMT-585	*srSm* RNA	AGCTAGGGCTTTTCCGTTG	5′RACE-PCR
CMT-180	Poly-G tail	GAATTCGAATTC CCCCCCCCCCC	5′ RACE-PCR
CMT-181	Poly-T tail	GAATTCGAATTC AAAAAAAAAAAA	5′ RACE-PCR
CMT-584	*fst-Sm*	GAATTC AGCCATTCAGAAAATAGCGC	5′ RACE-PCR
CMT-586	*srSm*	GAATTC GGGCTTTTCCGTTGCCAAA	5′ RACE-PCR
CMT-497	*fst-Sm*	ATGTGGCATAATTTCTTTATATA	Northern blot
CMT-498	*fst-Sm*	TTAATCGTCGTCTTTCTTATCC	Northern blot
CMT-572	*srfSm*	/5BiosG/AGCTAGGGCTTTTCCGTTGCCA	Northern blot
CMT-558	*srfSm*	/5BiosG/ATATTAAGGCATGATTTCTTTAT	Northern blot
CMT-672	5S rRNA	CCTAGGGGAGACACCTGT	Northern blot
CMT-673	5S rRNA	AGCTAAACTCCCTCTTGCT	Northern blot

a Restriction sites are underlined.

b Modified residues are shown in bold and underlined.

#### (i) Construction of expression vectors for induction in *E. coli*

To generate inducible expression constructs for induction of *fst-Sm* and *fst-Sm/srSm* in *E. coli*, fragments containing the open reading frame of *fst-Sm* and *fst-Sm/srSm* locus were first PCR amplified using UA159 genomic DNA (gDNA) as a template and the primer pairs CMT-499/CMT-500 for *fst-Sm* gene and CMT-581/CMT-582 for *fst-Sm*/*srSm* locus. The PCR products were purified, cloned in-frame upstream from the His_6_ sequence under the control of *araBAD* promoter into pBAD202/D-TOPO vector (Invitrogen), and transferred into *E. coli* TOP10. The recombinant plasmids were sequenced on both strands for confirmation. The plasmids designated pSK1 (*fst-Sm* in pBAD) and pSK2 (*fst-Sm/srSm* in pBAD) were then transferred into electrocompetent *E. coli* LMG194 cells for induction by arabinose.

#### (ii) Construction of a non-toxic Fst peptide

A non-toxic Fst peptide was generated by site-directed mutagenesis using the QuikChange II Site-Directed Mutagenesis Kit (Agilent Technologies) following the manufacturer’s recommendations. Briefly, plasmid pSK1 carrying the wild-type copy of *fst-Sm* gene was used as a template for the replacement of G16A using the two mutagenic PCR primers CMT-621 and CMT-622. The clone used to produce Fst-Sm G16A mutant was designated pSK7 and was confirmed by sequencing. The clone pSK7 was next used as a template with the two mutagenic PCR primers CMT-627 and CMT-628 for the replacement of A11G. The clone used for production of Fst-Sm G16A A11G mutant was designated pSK8 and was confirmed by sequencing. The clone pSK8 was then electrotransferred into LMG194 cells for induction by arabinose to produce the recombinant non-toxic Fst peptide (rNT-Fst).

#### (iii) Construction of GFP reporter constructs

First, a promoterless GFP vector was constructed by PCR amplifying the promoterless *gfp* cassette flanked by its upstream and downstream terminator sequences using pPROBE-NT’ [32] as template and the primer pair CMT-449/CMT-450. The PCR product was purified, double digested with XhoI/SacII, and then cloned into the shuttle vector pIB166 [33] pre-cut by the same enzymes. The recombinant plasmid pSK3 was confirmed by restriction digestion. To construct the *fst-Sm* promoter reporter fusions (P*_fst_*+DRI/II and P*_fst_*), the promoter region of *fst-Sm* with and without the direct repeats (DRI+DRII) was PCR amplified using UA159 gDNA as template and the primer pairs CMT-596/CMT-597 and CMT-596/CMT-598, respectively. The PCR products were double digested with EcoRI/KpnI and cloned into pSK3 precut with the same enzymes to generate the recombinant plasmids pSK4 (P*_fst_*+DRI/II) and pSK5 (P*_fst_*). To construct the *srSm* RNA expression vector, the *srSm* locus was PCR amplified using UA159 gDNA and the primer pair CMT-599/CMT-600. The PCR product was double digested with BamHI/EcoRI, purified, and cloned into pHGS299 precut by the same enzymes. The recombinant plasmid pSK6 was confirmed by sequencing.

#### (iv) Construction of vectors for expression in *S. mutans*

The full-length coding region and promoter region of *fst-Sm* and the *fst-Sm/srSm* locus were PCR amplified using UA159 gDNA as a template and the primer pairs CMT-596/CMT-595 and CMT-596/CMT-594, respectively. The PCR products were purified, double digested with EcoRI/HindIII, and cloned into pIB166 precut with the same enzymes. The recombinant plasmids pSK9 (*fst-Sm* in pIB166) and pSK10 (*fst-Sm/srSm* in pIB166) were sequenced on both strands for verification.

### RT-PCR

To confirm transcription of the putative *fst-Sm* toxin, reverse transcription-PCR (RT-PCR) was performed using the primers CMT-497 and CMT-498 (Table 2). Total RNA was isolated from UA159 WT strain cultures at early log (OD_600_ ∼ 0.1), mid-log (OD_600_ ∼ 0.5), and early stationary (OD_600_ ∼ 1.5) phases using TRIzol reagent (Invitrogen), DNase treated with RQ1 DNase (Promega), and converted to cDNA by using a RevertAid H Minus First Strand cDNA Synthesis Kit (Fermentas). Negative controls without reverse transcriptase enzyme were included in all experiments. As a positive control, the same RT-PCR primers were used to directly PCR amplify the UA159 gDNA. PCR products of 99 bp were resolved on a 2% (wt/vol) agarose gel.

### 5′RACE-PCR

A 5′ rapid amplification of cDNA ends (5′RACE)-PCR technique was used to define the transcriptional start site of *fst-Sm* mRNA and *srSm* RNA. Primers used for the 5′RACE-PCR assays are listed in Table 2. The assays were carried out with total RNA isolated from mid-log phase (OD_600_ ∼ 0.5) UA159 WT cultures by using TRIzol reagent (Invitrogen). DNA-free RNA (5 µg) was reverse transcribed by using the RACE outer primers CMT-583 and CMT-585 for *fst-Sm* gene and *srSm* RNA, respectively, and RevertAid H Minus First Strand cDNA Synthesis Kit (Fermentas) according to supplier’s instructions. RNase H and RNase T1 (Ambion) were then added, followed by incubation at 37°C for 30 min. The cDNAs were purified by using a GeneJET PCR Purification Kit (Fermentas) according to the manufacturer’s instructions. Tailing of purified cDNA was performed using the terminal deoxynucleotidyl transferase (Invitrogen) and dGTP/dTTP according to the manufacturer’s instructions. Tailed cDNAs were then PCR amplified by using the RACE universal primers CMT-180 (Poly-G tail) and CMT-181 (Poly-T tail), and the RACE inner primers CMT-584 (*fst-Sm* gene) and CMT-586 (*srSm* RNA). The amplicons were analyzed by agarose gel electrophoresis, and sequenced using the RACE inner primers CMT-584 and CMT-586.

### Northern Blot Analysis

Total RNA was isolated from UA159 WT strain cultures at early log (OD_600_ ∼ 0.1), mid-log (OD_600_ ∼ 0.5), early stationary (OD_600_ ∼ 1.5), and late stationary (OD_600_ ∼ 1.1) phases using TRIzol reagent (Invitrogen). Ten micrograms of total RNA was loaded by lane and resolved on a 12% (wt/vol) polyacrylamide denaturing gel containing 8 M urea. Size-fractionated RNA was transferred to a positively charged nylon membrane (Roche) using a Bio-Rad Mini Trans-Blot Cell and subjected to UV cross-linking. Membranes were pre-hybridized for 30 min at 42°C and followed by hybridization with biotin-labeled DNA probes (1 ng/ml for PCR probe and 10 ng/ml for oligoprobes) in ULTRAhyb hybridization buffer (Ambion) at 42°C overnight. The *fst-Sm* probe (99 bp DNA fragment) was PCR amplified from UA159 gDNA using CMT-497 and CMT-498 primers (Table 2) and labeled with Psoralen-biotin using a BrightStar Psoralen-Biotin Nonisotopic Labeling Kit (Ambion) according to supplier’s instructions. The biotin-labeled DNA oligoprobes CMT-558 and CMT-572 (Table 2) directed against the 5′ and 3′ UTR of *srSm* RNA, respectively, were purchased from Integrated DNA Technologies (Coralville, IA). The BrightStar BioDetect Kit (Ambion) was used for the detection of *fst-Sm* mRNA and *srSm* RNA following the manufacturer’s instructions.

### RNA Half-life Determination

An overnight culture of *S. mutans* UA159 WT strain was diluted (1∶20) into fresh THYE broth and incubated at 37°C until mid-log phase (OD_600_ ∼ 0.5) was reached. Rifampicin was added to a final concentration of 300 µg/ml and total RNA was isolated from 20-ml aliquots of culture taken at 0, 2, 5, 10, 15, 20, 40, 60, 90, 120, and 150 min after the addition of rifampicin. Northern blotting was performed as described above. For the detection of *fst-Sm* mRNA and 5S rRNA, probes were PCR amplified from UA159 gDNA using the primer pairs CMT-497/CMT-498 and CMT-672/CMT-673, respectively, and labeled with Psoralen-biotin using a BrightStar Psoralen-Biotin Nonisotopic Labeling Kit (Ambion) according to supplier’s instructions. For the detection of *srSm* RNA, the biotin-labeled DNA oligoprobe CMT-558 purchased from Integrated DNA Technologies (Coralville, IA) was used. Quantification of the signal was performed using the ChemiDoc XRS System and Quantity One software provided by Bio-Rad.

### Toxicity Assays in *E. coli*

Overnight cultures of LMG194(pSK1), LMG194(pSK2), and LMG194(pSK8) were diluted (1∶100) into fresh LB medium supplemented with kanamycin at 50 µg/ml and grown aerobically at 37°C. At an OD_600_ of ∼0.5, 0.2% (wt/vol) glucose (uninduced control) or 0.002% (wt/vol) arabinose was added to induce the expression of recombinant fusion proteins. Protein expression was verified by SDS-PAGE using the buffer system of Laemmli. The protein bands were visualized by staining with Coomassie brilliant blue. Aliquots of cultures were removed at 0, 15 min, 30 min, 45 min, 1 h, 2 h, and 3 h post-induction, serially diluted and plated on LB agar plates. Colonies were counted after 16 h of incubation at 37°C. All experiments were performed in triplicate from three independent experiments.

### Toxicity Assays in *S. mutans*

Overnight cultures of *S. mutans* were diluted (1∶20) into fresh THYE broth and grown at 37°C until an OD_600_ of ∼0.1 was reached. Cultures were then divided into 0.5-ml aliquots: (i) pIB166 (control), (ii) pSK9 (*fts-Sm* in pIB166), (iii) pSK10 (*fts-Sm/srSm* in pIB166). Portions (1 µg) of plasmids were added to the cultures, which were grown for a further 2.5 h at 37°C in the presence of synthetic competence-stimulating peptide at a final concentration of 0.5 µg/ml. Cultures were serially diluted and plated on THYE agar plates. The transformation efficiency was calculated as the percentage of chloramphenicol-resistant transformants divided by the total number of recipient cells, which was determined by the number of CFU on antibiotic-free THYE agar plates. All assays were performed in triplicate from three independent experiments.

### GFP-based Assay

Overnight cultures of *E. coli* strains harboring the GFP reporter constructs were diluted (1∶100) into fresh LB broth supplemented with chloramphenicol and kanamycin. Cells were grown aerobically at 37°C until an OD_600_ of ∼0.4 was reached (∼ 6×10^7^ CFU/ml). Cells (2-ml aliquots) were harvested by centrifugation, washed once with sterile phosphate-buffered saline (PBS), and resuspended in 1 ml of sterile PBS. Green protein fluorescence was detected in terms of relative fluorescence units (RFU) with a fluorescence spectrophotometer. The excitation and emission filters were 488 nm and 511 nm. All experiments were performed in triplicate from two independent experiments.

### Persistence Assay

Overnight cultures of *S. mutans* (WT vs. ΔIGR176; WT(pIB166) vs. ΔIGR176(pSK10)) were diluted (1∶20) into fresh THYE broth containing oxacillin (2 µg/ml), cefotaxime (2 µg/ml), or vancomycin (20 µg/ml) and incubated for 24 h at 37°C. Aliquots were removed at the indicated times, serially diluted, and plated on THYE agar plates. The colonies were counted after 48 h of incubation. All assays were performed in triplicate from three independent experiments.

## Results

### Detection of RNAs from the Intergenic Region IGR176

**Figure 1:**
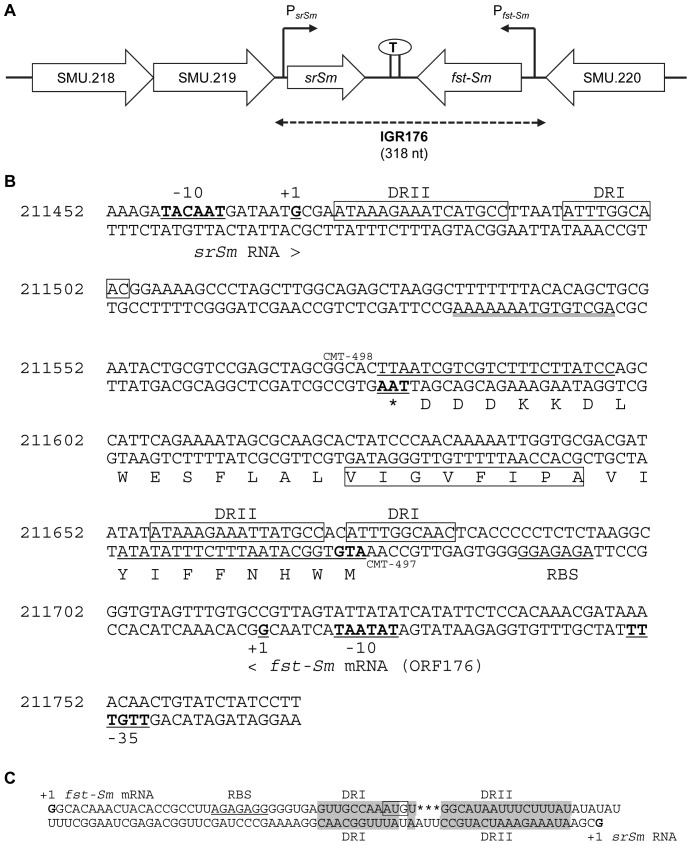
Analysis of *S. mutans* IGR176 region. (**A**) Schematic representation of the location of the *fst-Sm*/*srSm* locus on the *S. mutans* chromosome. Arrows indicate the direction of transcription. The *fst-Sm* and *srSm* promoter sequences are indicated by P*_fst-Sm_* and P*_srSm_*, respectively. A predicted stem-loop bidirectional terminator is indicated between *srSm* and *fst-Sm*. Shown at the bottom are the boundaries of the intergenic region IGR176. (**B**) Nucleotide and amino acid sequences of the *S. mutans fst-Sm*/*srSm* type I TA locus located in the intergenic region IGR176 (from 211452 to 211769) of UA159 genome. The conserved APUU(A/V)GUU motif present in Fst-Sm peptide is boxed. Putative promoter sites of *fst-Sm* toxin (–35, –10) and *srSm* antitoxin (–10), ribosome binding site (RBS) of Fst-Sm toxin, and a factor-independent bidirectional terminator (double underlined) are indicated. The transcriptional start site (+1) of *fst-Sm* and *srSm* identified by 5′ RACE-PCR are indicated below the sequence. The regions encoding the DRI and DRII repeats are boxed. The primers CMT-497 and CMT-498 used in the RT-PCR experiments are underlined. (**C**) Proposed RNA:RNA interactions (in shaded regions) between *fst-Sm* mRNA and *srSm* RNA.

A study by Weaver et al. (2009) [30] previously identified pAD1-like TA systems on the chromosomes and plasmids of *Enterococcus*, *Lactobacillus*, and *Staphylococcus* species suggesting that Fst-like toxins may be prevalent in Gram-positive bacteria. Recently, an exhaustive PSI-BLAST and TBLAST searches across 774 bacterial genomes identified several homologs of type I toxins [4], . Analysis of the genome of *S. mutans* UA159 reference strain predicted a putative type I toxin belonging to the Fst family in the intergenic region IGR176. This small intergenic region (318 nt) is located between the genes SMU.219 and SMU.220 (Fig. 1A) encoding a Zn-dependent protease of the COG2856 family [8] and a hypothetical protein of unknown function, respectively. SMU.219 is mostly co-transcribed with an upstream gene, SMU.218, encoding a HTH domain-containing protein of the Xre family. The SMU.218/SMU.219 gene pair is predicted to function as *bona fide* type II TA system (TADB database: http://bioinfo-mml.sjtu.edu.cn/TADB/) [34].

Bioinformatic analysis revealed that the intergenic region IGR176 contains an unannotated open reading frame of 99 bp, which we named ORF176 (Fig. 1B). ORF176 encodes a putative peptide of 32 amino acid residues with a predicted MW of 3613.3 Da. The putative peptide showed homology to the Fst toxin family [4], [5]. The APUU(A/V)GUU motif (where U equals Ile, Leu, Val, or Phe) present in the hydrophobic region of the Fst family of RNA-regulated peptides [30] was also identified in the putative translated ORF176 peptide. We first determined whether or not an mRNA was transcribed by RT-PCR using gene-specific primers and RNA isolated from *S. mutans* WT cells during early-log, mid-log, and early stationary phases. A single PCR product of 99-bp corresponding to ORF176 was detected in all growth phases; the identity of the amplicon was confirmed by DNA sequencing (data not shown). To map the 5′ end of ORF176, we performed 5′RACE-PCR. The apparent start site (+1) was located 7 nucleotides 3′ proximal to a canonical –10 sequence (TATAAT). An obvious –35 sequence (TTGTTT) is also present spaced by 21 nucleotides from the –10 box (Fig. 1B). Taken together, these results provide genetic evidence for the presence of a previously unannotated *fst*-like toxin gene in the intergenic region IGR176 of *S. mutans*. We proposed the name *fst-Sm* to designate the gene encoding a putative Fst-like toxin in *S. mutans*, a predicted type I toxin.

We next hypothesized that the putative Fst-Sm toxin could be regulated post-transcriptionally by small RNA base pairing. Therefore, we performed a visual inspection of the DNA sequence immediately surrounding the *fst-Sm* toxin gene and searched for a sequence that would generate an RNA that could potentially bind the *fst-Sm* mRNA. A putative small RNA, which we named *srSm* (small RNA *S. mutans*), was found directly opposite the coding sequence of *fst-Sm* (Fig. 1B). The sequence was predicted to encode a small untranslated RNA as it contains potential –35 and –10 promoter elements but no open reading frame (ATG start codon) or ribosome binding site (RBS). To test further whether *srSm* is an untranslated RNA, we searched for open reading frames preceded with an alternative translational start codon (GTG or TTG). The fact that the longest open reading frame was only 10 codons in length (using TTG as putative initiation codon) and no putative RBS could be identified, suggest that *srSm* RNA is not translated. Using 5′RACE-PCR, we next determined the transcriptional start site of *srSm*. The *srSm* RNA starts at the G position, 108 bp upstream the *fst-Sm* stop codon (Fig. 1B) confirming transcription of *srSm* RNA. Moreover, the 5′ end mapping results indicate that both *fst-Sm* mRNA and *srSm* RNA are transcribed across direct tandem repeats, DRI and DRII (Fig. 1B), at which interactions mostly occur. The direct repeats overlap the start codon of *fst-Sm* mRNA leading most likely to suppression of translation.

**Figure 2:**
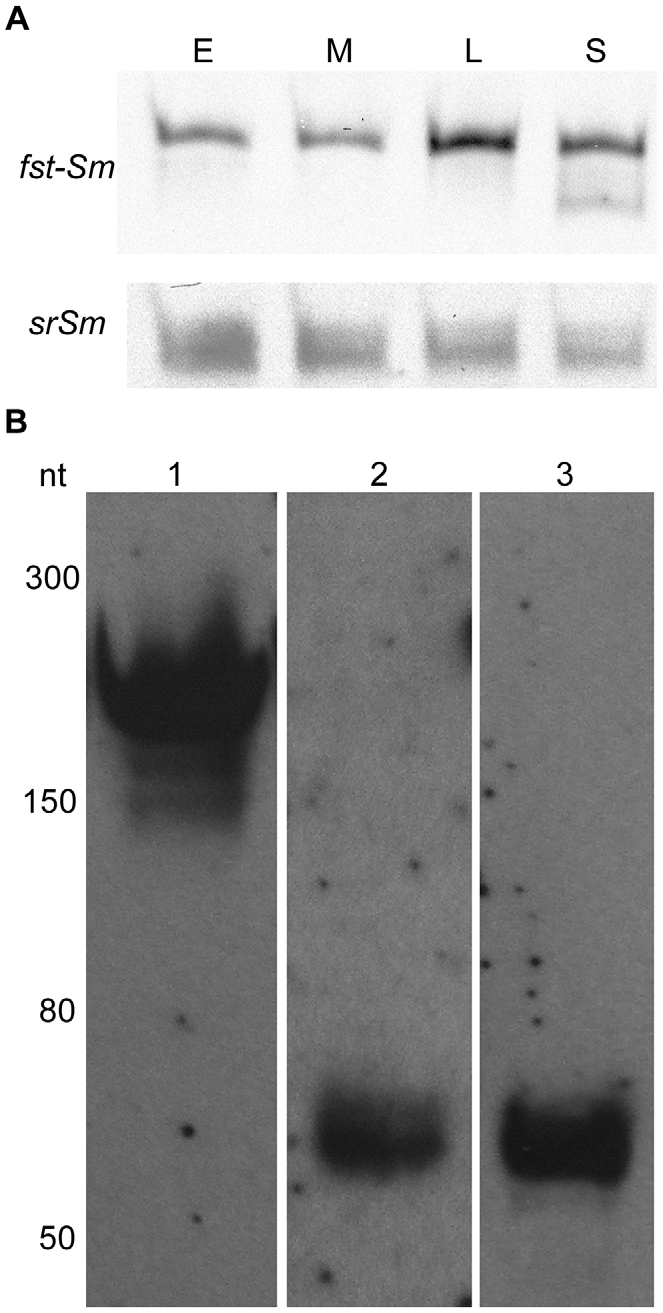
Detection of RNAs in IGR176 by Northern blot analysis using biotin-labeled DNA probes. Total RNA from *S. mutans* WT strain was resolved on a 12% polyacrylamide denaturing gel containing 8 M urea. (**A**) Total RNA was extracted from UA159 WT cells during the early log (E), mid-log (M), late log (L), and stationary (S) phase of growth. The blot of *fst-Sm* was probed with a PCR-amplified double-stranded DNA that corresponds to the full-length coding region, while the blot of *srSm* was probed with CMT-558 DNA oligoprobe. The blots shown are representative of three independent experiments. (**B**) Total RNA isolated from UA159 WT cells grown to mid-log phase. *fst-Sm* mRNA was detected with a PCR-amplified double-stranded DNA that corresponds to the full-length coding region (lane 1). Oligoprobes CMT-558 (lane 2) and CMT-572 (lane 3) corresponding to the 5′ and 3′ UTR of *srSm*, respectively, were used to detect the *srSm* RNA. Low Range ssRNA Ladder (New England Biolabs) are indicated in nucleotides on the left.

Northern blotting was next performed to investigate expression of *fst-Sm* gene and *srSm* RNA during growth, and to determine the sizes of the transcripts produced from the *fst-Sm*/*srSm* locus. As shown in Fig. 2A, transcripts specific to *fst-Sm* and *srSm* RNAs were detected throughout *S. mutans* growth under nutrient-rich conditions. Interestingly, a smaller transcript was also observed for *fst-Sm* gene during late stationary growth phase suggesting that specific processing may also occur. Transcripts of ∼200 nt and ∼70 nt specific to *fst-Sm* mRNA and *srSm* RNA, respectively, were detected by Northern blot analysis (Fig. 2B). The signals obtained are in agreement with the predicted size of the RNAs inferred from the experimentally mapped +1 transcriptional start site of *fst-Sm* and *srSm* RNAs. Altogether, these results confirm that the intergenic region IGR176 expresses the *fst-Sm* gene, a previously unannotated open reading frame, and a *cis*-encoded small RNA. Consequently, the *S. mutans fst-Sm*/*srSm* locus encodes a predicted type I TA system.

### Stability of the *fst-Sm* and *srSm* RNAs

**Figure 3:**
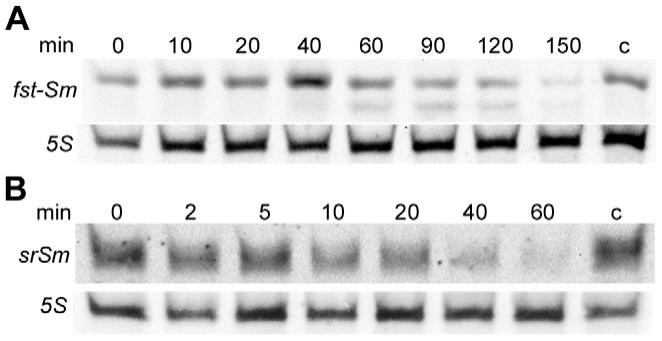
RNA half-life determination. Stability of *fst-Sm* mRNA (**A**) and *srSm* RNA (**B**) by Northern blot analysis. Total RNA was extracted from WT mid-log cells at the indicated times after addition of 300 µg/ml rifampicin. Time points of sampling are indicated above each lane. Biotin-labeled DNA probes were used for RNA detection. The probing for 5S RNA confirmed equal loading. Control RNA extraction represents total RNA extracted from cells cultivated without rifampicin at time-point 150-min (*fst*-*Sm* mRNA detection) and 60-min (*srSm* RNA detection). Blots shown represent results from three experiments.

The relative stabilities of both the toxin mRNA and antitoxin small RNA are an important aspect of TA regulation. In TA systems, the antitoxin is less stable than the toxin in the cell, and consequently it has to be constantly produced to inhibit the toxin [3], [35]. In order to test whether *srSm* RNA is less stable and might therefore represent an antisense RNA antitoxin, we determined the half-life of *fst*-*Sm* mRNA and *srSm* RNA by Northern blotting. WT strain was cultivated in THYE medium until mid-log growth phase was reached. Rifampicin was then added to prevent the initiation of RNA synthesis, time samples were taken, and RNA was extracted and subjected to Northern blot analysis. The half-life of *srSm* RNA was determined to be ∼ 30 min, while *fst*-*Sm* mRNA had a half-life of ∼ 90 min under the conditions tested (Fig. 3). Antisense RNAs of bacterial plasmids generally exhibit very short half-lives (<2 min), whereas long half-lives (20–60 min) were reported for regulatory sRNAs encoded by bacterial chromosomes [36], [37]. Northern blot analyses of *fst-Sm* transcript also indicated the presence of another RNA size in addition to the full-length transcript (Fig. 3A) suggesting that *fst-Sm* mRNA undergoes specific processing, most likely affecting its RNA stability. These data indicate that the *fst-Sm* toxin mRNA is much more stable than its *srSm* antitoxin RNA. These results are consistent with the findings of previous studies on other type I TA systems [3], [35].

### *srSm* RNA Expressed in *Trans* Represses GFP Expression

**Figure 4:**
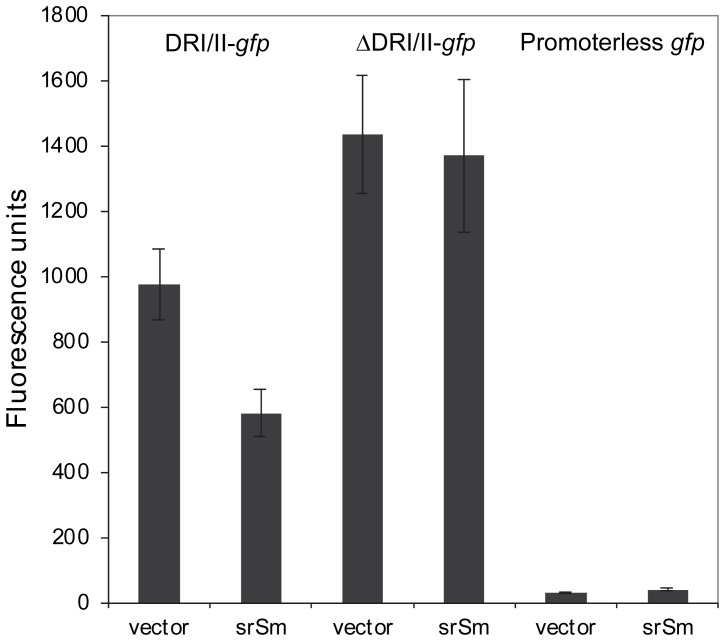
The *srSm* RNA represses GFP expression. Fluorescence from plasmid-encoded DRI/II-*gfp* (pSK4) or ΔDRI/II-*gfp* (pSK5) transcriptional fusions was measured in *E. coli* DH10B carrying pHSG299 vector expressing *srSm* (pSK6) or the empty vector. The promoterless GFP vector (pSK3) was used as negative control. All experiments were performed in triplicate from two independent experiments. The means ± SDs are shown.

Our results suggest that *fst-Sm*/*srSm* locus encodes a type I TA system, in which *srSm* RNA antitoxin functions through an antisense base-pairing mechanism that results in inhibition of toxin translation. As both *fst-Sm* and *srSm* RNAs are transcribed across the direct tandem repeats DRI and DRII (5′ end mapping results, Fig. 1B), we hypothesized that *srSm* RNA (antisense) could bind to the DRI/DRII region in *fst-Sm* mRNA (sense) thereby controlling its expression (Fig. 1C). To test this hypothesis, we designed a two-plasmid gene reporter system to investigate whether the *srSm* RNA acts on the *fst-Sm* mRNA. The promoter of *fst-Sm* and its 5′ mRNA coding region with the DRI and DRII repeats were transcriptionally fused to *gfp* gene into the promoterless-GFP plasmid pSK3. The resulting recombinant plasmid, pSK4, was then introduced into an *E. coli* strain carrying the pHGS299 vector expressing *srSm* RNA (pSK6). Cells were grown until mid-log phase and fluorescence was measured. We reasoned that, if *srSm* repressed GFP expression in this *E. coli* strain, it would indicate that *srSm* RNA acts directly on the *fst-Sm* mRNA. Indeed, when *srSm* was introduced in *E. coli*(pSK4), approximately two-fold repression of GFP occurred (Fig. 4). We next cloned the promoter of *fst-Sm* and its 5′ mRNA coding region without the DRI/II repeats in frame with *gfp*. The recombinant plasmid, pSK5, was then introduced into *E. coli*(pSK6) and fluorescence was measured. No repression was observed compared with an *E. coli* strain containing the empty vector (Fig. 4). Based on the above data, we can predict that *srSm* directly binds to the DRI/II repeats to inhibit Fst-Sm expression.

### Induced *fst-Sm* Expression Confers Toxicity in *E. coli*

**Figure 5:**
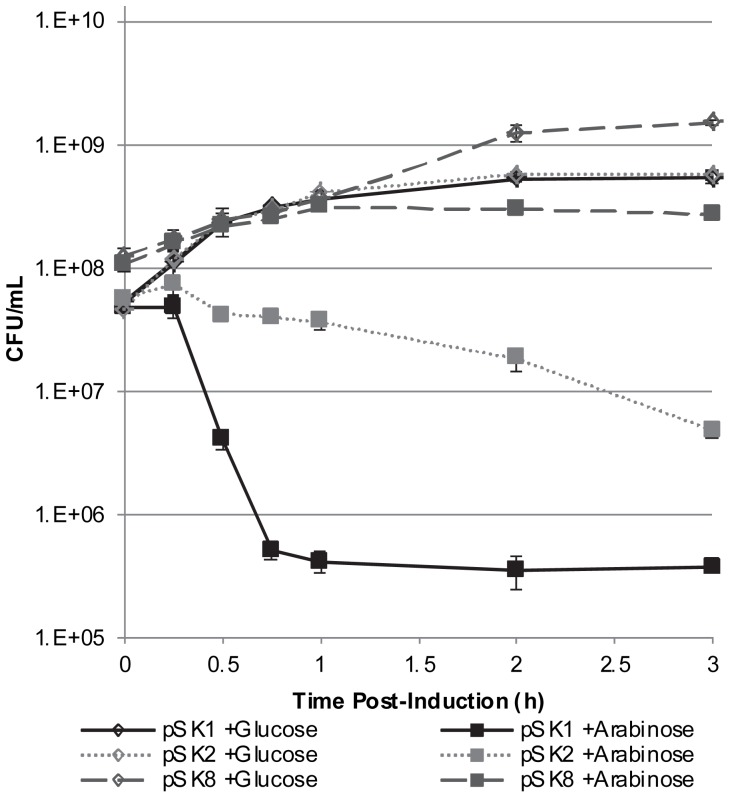
Characterization of the Fst-Sm/srSm TA system in *E. coli*. Cells of LMG194 containing pSK1 (Fst-Sm), pSK2 (Fst-Sm/srSm), and pSK8 (NT-Fst) were grown to mid-log phase, at which time arabinose (induced) and glucose (uninduced control) were added. After induction, appropriate dilutions were plated on LB agar for determination of the number of CFU per ml. The curves presented are the averages and standard deviations of results from three independent cultures.

The *S. mutans fst-Sm*/*srSm* locus was next investigated to determine whether it constitutes a functional type I TA system. The *fst*-*Sm* gene was cloned into the pBAD expression system for induction of gene expression using *araBAD* promoter dose-dependent regulation. The recombinant plasmid designated pSK1 was used to transform *E. coli* LMG194 strain. Overexpression of Fst-Sm resulted in inhibition of colony formation (data not shown). In cell viability assays, growth of *E. coli* was markedly affected when Fst-Sm was overexpressed from the *araBAD* promoter (Fig. 5). Overexpression of Fst-Sm resulted in a reduction of cell viability of ∼ 90% and ∼ 99% by 30 min and 45 min post-induction, respectively. By contrast, LMG194(pSK1) cultivated in presence of glucose (uninduced control) did not show any growth defects (Fig. 5). This indicated that Fst-Sm is a toxin. Furthermore, we constructed a plasmid for arabinose-inducible overexpression of a mutated Fst-Sm toxin. Single-site mutations of amino acid residues were introduced by two rounds of PCR in the conserved hydrophobic sequence of Fst toxin since this region of Fst has been shown to be important for Fst toxicity [30]. First, the conserved glycine at position 16 was changed to alanine. The alanine mutant (G16A) was then used to mutate alanine to glycine at position 11. The resulting pSK8 construction (G16A, A11G) was finally transferred to LMG194 strain and tested using our cell viability assay. As shown in Fig. 5, LMG194(pSK8) cultivated in liquid LB medium supplemented with arabinose did not present any growth defects and behaved similarly to the control curve (uninduced glucose control). These results highlight the importance of this conserved hydrophobic region in Fst-Sm toxicity.

We next measured the toxicity of Fst-Sm peptide when overexpressed in pBAD plasmid with its *cis*-encoded *srSm* RNA under the control of its own promoter. The effect of overexpressing Fst-Sm/srSm on growth was significantly less (∼ 25% reduced cell viability by 30 min post-induction), but Fst-Sm still affected growth (∼ 90% reduced cell viability by 3 h post-induction) most probably due to insufficient levels of *srSm* RNA transcribed from its own promoter (Fig. 5). Based on these results, we can conclude that Fst-Sm confers toxicity to *E. coli* and expression of its *cis*-encoded *srSm* is able to curb Fst toxicity.

### Overexpression of *fst-Sm* is Lethal to *S. mutans* Cells

We next measured the toxicity of Fst-Sm toxin in *S. mutans*. We designed our toxicity assay based on the natural transformation ability of *S. mutans* [23]. The individual *fst-Sm* gene and the *fst-Sm*/*srSm* locus were placed under the control of the constitutive P*_23_* promoter into the plasmid pIB166. Overnight cultures of *S. mutans* WT strain and its ΔIGR176 mutant were diluted in fresh medium and grown to early log phase prior to addition of pIB166 (empty plasmid), pSK9 (*fst-Sm* in pIB166), or pSK10 (*fst-Sm*/*srSm* in pIB166). Cells were grown for a further 2.5 h before differential plating. Our results showed that WT and ΔIGR176 mutant cells were readily transformed with pIB166 and pIB166 bearing *fst-Sm*/*srSm* locus. Indeed, the transformation efficiencies of WT and ΔIGR176 mutant for the empty vector were similar to the efficiencies obtained with the pSK10 construct (Table 3). This was not the case when the pSK9 construct was transferred to WT strain and ΔIGR176 mutant. The transformation efficiency of the WT strain was reduced by more than 1,000-fold for pSK9 bearing the *fst-Sm* toxin gene only, corresponding to less than 0.07% of the pIB166 control value. For ΔIGR176 mutant, less than 5 colonies were obtained when undiluted cells were plated (Table 3). The fact that the transformation efficiency was much higher for WT strain compared with the ΔIGR176 mutant for pSK9 (almost no colonies could be detected for ΔIGR176 after 48 h of incubation) suggested that the chromosomal copy of the *srSm* RNA (*trans*-encoded) in WT strain might confer some protective effects. These results clearly showed a growth-inhibitory effect of Fst-Sm on *S. mutans* cells and that the toxic effect elicited by Fst-Sm can be neutralized by coexpression of its *srSm* RNA. Hence, *fst-Sm*/*srSm* encodes a functional type I TA system in *S. mutans*.

**Table 3:** *S. mutans* toxicity assay based on natural competence.

Construct	Description	Transformation Efficiency ± SD^a^	
		WT	ΔIGR176
pIB166	Empty vector	(1.1±0.6) ×10^0^	(2.2±0.6) ×10^−2^
pSK9	*fst-Sm* gene	(8.5±1.5) ×10^−4^ *	<5 colonies ^¶^
pSK10	*fst-Sm*/*srSm* locus	(7.0±0.4) ×10^−1^	(4.0±0.7) ×10^−3^

a The transformation efficiency was expressed as the percentage of chloramphenicol-resistant transformants divided by the total number of recipient cells. All experiments were performed in triplicate from three independent experiments. Statistical significance: * WT(pSK9) vs. WT(pIB166); ^¶^ΔIGR176(pSK9) vs. ΔIGR176(pIB166).

### Deletion and Mild-overexpression of IGR176 Region has no Effect on *S. mutans* Cell Growth

To study the cellular function of Fst-Sm/srSm system, we first investigated microbial growth kinetics of WT, ΔIGR176 mutant, and IGR176 complemented strains. The absence of Fst-Sm/srSm TA system or its mild-overexpression did not affect cell growth in liquid medium under the conditions tested (data not shown). No effect on cell growth was observed when *S. mutans* cells were exposed to extracellular recombinant Fst-Sm peptide at concentration up to 160 µg/ml. We next conducted a long-term survival assay using monocultures of WT and its ΔIGR176 mutant cultivated in a nutrient-rich medium (THYE) or a chemically defined medium (CDM). After 14 days, there was no difference in survival between WT and mutant strains under both nutrient growth conditions (data not shown). Biofilm formation was investigated using static biofilms developed in polystyrene microtiter plates. Assessment of the biofilm-forming capacity of ΔIGR176 mutant and IGR176 complemented strain relative to WT showed that both mutants formed stable and reproducible biofilms typical of the parent strain in ¼-THYE-sucrose and ¼-THYE-glucose. No significant differences in the total biomass of the mutant biofilms compared with the WT biofilms were noted for 24-h-, 48-h-, and 72-h-old biofilms (data not shown).

### Ectopic Expression of Fst-Sm/srSm Type I TA Module Decreases Persister Cell Formation in *S. mutans*

**Figure 6:**
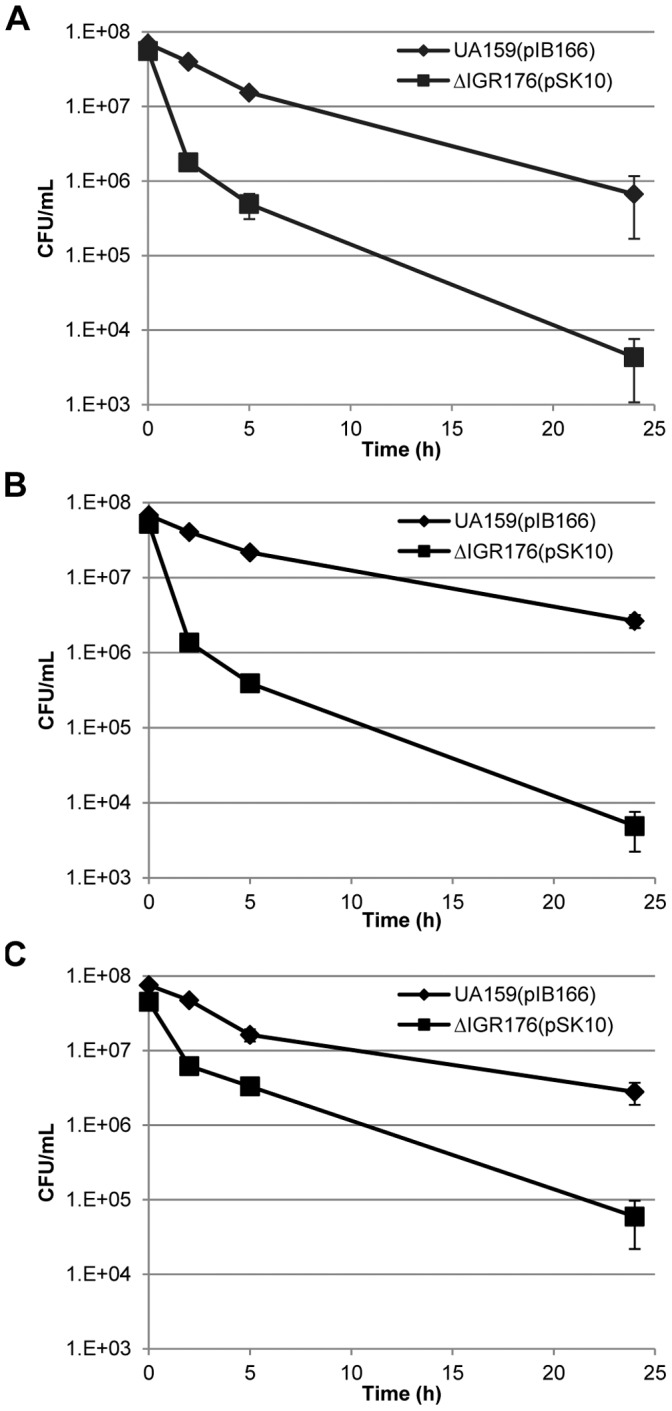
Effects of mild-overexpression of Fst-Sm/srSm type I TA system on *S. mutans* persister formation. Oxacillin-treated (**A**), cefotaxime-treated (**B**), and vancomycin-treated (**C**) cells were removed at the indicated time points, serially diluted, spot plated onto THYE agar plates, and the number of CFU per ml was determined from plate counts. The curves presented are the averages and standard deviations of results from three independent cultures.

Persister cells are phenotypic variants that are extremely tolerant to high concentrations of antibiotics. They make up a small part of the bacterial population [38]. In *S. mutans*, type II TA systems have been linked to persister formation. Indeed, mild-overexpression of the MazEF and RelBE type II TA modules resulted in an increase in multidrug-tolerant persister levels [24]. To examine whether the Fst-Sm/srSm type I TA system was also involved in persister cell formation, we first tested an IGR176 knockout mutant. As expected, deletion of Fst-Sm/srSm system did not affect the persister levels after challenge with a high dose of the cell wall synthesis inhibitors oxacillin, vancomycin, and cefotaxime (data not shown) due most likely to redundant pathways for bacterial persister formation. Similar results were obtained using the Δ*mazEF* and Δ*relBE* deletion mutants [24], reinforcing the hypothesis that more than one single mechanism is responsible for persister formation. In contrast, the ectopic expression of Fst-Sm/srSm module caused a dramatic decrease in the number of persister cells. Our data showed that overexpression of Fst-Sm/srSm system decreased the number of oxacillin-tolerant (∼145-fold decrease at 24 h), cefotaxime-tolerant (∼240-fold decrease at 24 h), and vancomycin-tolerant (∼50-fold decrease at 24 h) persisters (Fig. 6). The fact that the MIC values of oxacillin, cefotaxime, and vancomycin against ΔIGR176(pSK10) mutant were not different from those for the WT strain confirmed that the complemented strain was a true persister mutant.

## Discussion

TA systems are small genetic modules that are widespread in the prokaryotic kingdom [4], [8], [9]. There are of five different types depending of the nature and mode of action of the antitoxin component. The type III, IV, and V were discovered very recently and are represented so far by unique examples [6], [7], [11]–[13]. While type II TAs are highly represented in bacterial chromosomes, little is known about the distribution of type I TA loci. One possible reason could be that all the toxins of the type I TAs consist of very small hydrophobic peptides [3] and gene annotation software frequently fails to identify short protein-coding genes in microbial genomes [39]. Moreover, the development of computational approaches to identify novel type I TA systems is challenging owing to the short, hydrophobic character of the toxins, and the difficulty in predicting the antitoxin small RNAs [4]. In the present study, we reported to our knowledge the first functional type I TA system in streptococci. TAs are commonly described as constituents of the prokaryotic mobilome [4]. The chromosomal *fst-Sm*/*srSm* locus is located in a transposon-related genomic island of *S. mutans* UA159 strain suggesting that this type I TA module may have moved through horizontal gene transfer. IGR176 could not be found in other sequenced *S. mutans* genomes by searching public genomic sequence. This result was not surprising since transposon element movement is one of the major causes for intraspecies sequence variations in the intergenic regions. Collectively our results support that Fst-Sm/srSm forms a functional type I TA pair in the oral pathogen *S. mutans*. These results are: i) *fst-Sm* encodes a small hydrophobic peptide belonging to the Fst toxin family; ii) a *cis*-encoded small RNA was found converging towards the end of the toxin gene; iii) *srSm* antisense RNA is less stable than *fst-Sm* mRNA; iv) Fst-Sm has a toxic effect on *S. mutans* which can be neutralized by expression of *srSm* antitoxin; v) both *fts-Sm* mRNA and *srSm* RNA are transcribed across direct tandem repeats providing a region of complementarity between the *srSm* antisense RNA and the translation initiation region of *fst-Sm* mRNA; vi) *srSm* RNA most likely binds to the direct tandem repeats in the *fst-Sm* mRNA occluding Fst-Sm start codon. Although our results using the GFP constructs suggest that pairing of *srSm* RNA with the 5′ end of *fst-Sm* mRNA suppresses toxin translation, additional experiments will be required to determine if this pairing leads to translational silencing or mRNA degradation. It is also possible that degradation is secondary to translation inhibition [40], [41]. Indeed, the *srSm*:*fst-Sm* duplex is a good target for cleavage by RNase III, an endoribonuclease specific for double stranded RNA. Attempts to construct a *S. mutans* RNase III mutant were unsuccessful, suggesting that this may be an essential gene in *S. mutans*.

The toxic phenotype of many chromosomal type I toxins have been reported upon overexpression from a multicopy plasmid. So far all type I toxins characterized from *E. coli* are predicted to function through the same mechanism as phage holins, forming a pore to destroy the membrane potential and to inhibit ATP synthesis [42]–[45]. In *E. faecalis*, the intracellular overproduction of the plasmid-encoded Fst toxin compromised the integrity of the cell membrane and specific defects in chromosome partitioning and cell division were also observed [28], [29]. At present, the mechanism of action of Fst-Sm toxicity is not known. Our data suggest that Fst-Sm functions intracellularly since extracellular addition of recombinant Fst-Sm toxin to cells in cultures had no effect on growth of *S. mutans* and *E. coli*. Preliminary examination of Fst-induced *E. coli* cells by scanning electron microscopy showed no evidence of ghost cell formation or any other abnormal cellular morphology compared with the non-toxic Fst-induced cells (data not shown). Further experiments will be required to identify the primary target of Fts-Sm toxin in its native host. For instance, construction of a tightly regulated inducible expression system for *S. mutans* will be necessary to study the effects of Fst-Sm induction on *S. mutans* cellular morphology. It would also be interesting to investigate whether Fst-Sm toxin could affect cell division by inhibiting the polymerization of *S. mutans* cytoskeleton proteins (*e.g*., FtsQ, FtsA, FtsZ) in future studies.

The physiological function of chromosomal TA systems remains unclear. In fact, the functions of chromosomal TAs are diverse and may depend on the type of TA, its genomic location, and host species. Several roles have been discovered for type II TA systems, including stress survival, growth control, programmed cell death, persister formation, stabilization of the genome, biofilm formation, phage abortive infection, and anti-addiction system [1], [2], [15], [46]. We previously demonstrated that ectopic mild-overexpression of type II TA systems generated greater number of persister cells in *S. mutans*. The picture that emerged from our work and from several other studies is clearly pointing out to a multi-gene function and suggests that *S. mutans* did not evolve a dedicated mechanism allowing it to adopt a persistence phenotype [24]. Not surprisingly, the deletion of *fst-sm*/*srSm* locus did not affect the persister levels (data not shown). To date, the *E. coli* MqsR/MqsA type II TA is the only system that affects (decreases) persister formation upon deletion [47]. Our prediction was that mild-overexpression of Fst-Sm/srSm system would increase persister levels, promoting antibiotic tolerance. Quite to the contrary, when we overexpressed Fst-Sm/srSm system, we observed a decrease in persister levels surviving cell wall-acting antibiotic treatments. In a typical persister experiment, a biphasic killing curve is obtained consisting of an initial drop in viable cell counts (the antimicrobial susceptibility of the bulk of the population) followed by a surviving subpopulation of persisters that decreased slowly over time. The decrease in persister levels obtained when the Fst-Sm/srSm system is overexpressed could be related to persister cells awakening from dormancy. These persister cells that start to grow in the presence of the antibiotic are then killed more rapidly. Indeed, the second phase revealing the resuscitation rate of persisters produced by the overexpressing mutant has a steeper slope in comparison to the WT control curve. Assuming that persister cells could provide a reservoir of viable bacteria that can acquire resistance by random mutation or horizontal gene transfer [17], the results from this study reinforce the idea that TA systems represent an attractive target for designing new drugs that could kill persister cells that have woken up. The clinical implications of TA systems and persister cell formation in the context of chronic infections are thus highly significant.
